# Superfizielles Angiomyxom der Fußsohle

**DOI:** 10.1007/s00105-024-05395-8

**Published:** 2024-08-05

**Authors:** Silvia Mihalceanu, Wolfgang Hartschuh, Ferdinand Toberer

**Affiliations:** 1 Universitäts-Hautklinik Heidelberg, Ruprecht-Karls-Universität Heidelberg, Im Neuenheimer Feld 440, 69120 Heidelberg, Deutschland; 2 Praxis Dr. med. Durani, Grüne Meile 56, 69115 Heidelberg, Deutschland

**Keywords:** Kutanes Angiomyxom, Solitäres kutanes Myxom, Carney-Komplex, Histologie, Immunhistochemische Aufarbeitung, Cutaneous angiomyxoma, Solitary cutaneous myxoma, Carney complex, Histology, Immunohistochemistry workup

## Abstract

Das superfizielle Angiomyxom ist eine seltene, benigne Neoplasie, bestehend aus spindeligen Fibroblasten in einem myxoiden, gefäßreichen Stroma. Die diagnostische Abklärung gelingt meist mithilfe der histologischen und immunhistochemischen Aufarbeitung und ist in Abhängigkeit von der klinischen Präsentation von besonderem Stellenwert, da differenzialdiagnostisch auch maligne Tumoren infrage kommen. In unserer Fallvorstellung präsentieren wir einen Patienten mit einem solitären, superfiziellen Angiomyxom der Fußsohle. Obwohl das superfizielle Angiomyxom oft als eine eigenständige Entität auftritt, ist es empfehlenswert, Syndrome wie den Carney-Komplex auszuschließen.

## Abstract (en)

Superficial angiomyxoma is a rare, benign neoplasm consisting of spindle-shaped fibroblasts in a myxoid, vascularized stroma. Diagnostic clarification is usually based on histology and immunohistochemistry and is of particular importance depending on the clinical presentation, as malignant tumors may also be considered in the differential diagnosis. Herewith, we present a patient with a solitary, superficial angiomyxoma of the sole of the foot. Although it often occurs as an independent entity, it is recommended to exclude syndromes such as Carney complex.

## Anamnese

Ein 44-jähriger, sportlich aktiver Patient stellte sich mit einem Tumor am rechten medialen Fersenballen vor. Die Läsion war etwa 10 Jahre zuvor als eine eher weiche, indolente Schwellung erstmals aufgetreten. Der Patient berichtete über eine langsame Größenzunahme in den letzten Jahren mit Druckdolenz lediglich bei sportlicher Belastung (Laufen). Anamnestisch waren keine relevanten Vorerkrankungen bekannt, und der Patient nahm keine Dauermedikamente ein.

## Klinischer Befund

**Abb. 1:**
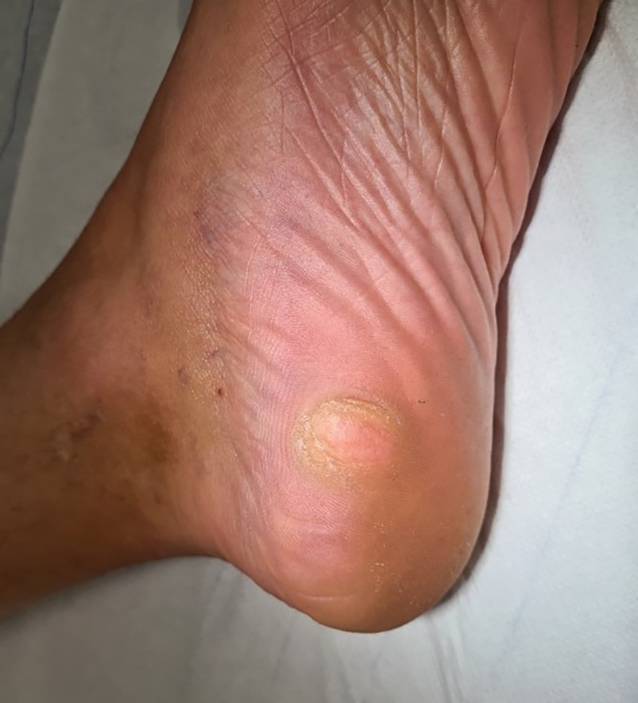
Rötlich durchscheinender Nodus am linken medialen Fersenballen

Bei der Inspektion zeigte sich am linken medialen Fersenballen ein 2,0 × 1,7 cm durchmessender, halbkugeliger Nodus. Dieser imponierte rötlich durchscheinend mit zentral glatter Oberfläche und deutlicher peripherer Hyperkeratose (Abb. 1). Palpatorisch ergab sich ein weicher bis prall-elastischer Tasteindruck. Das restliche Integument zeigte sich unauffällig.

Auswärts wurde die Verdachtsdiagnose eines Virusakanthoms gestellt. Unsererseits klinisch in Betracht gezogene Differenzialdiagnosen waren insbesondere piezogenes Knötchen, atypischer Naevus lipomatosus und ekkrines Porom. Da der Befund klinisch schwer einzuordnen war, wurde eine tiefe Messerbiopsie durchgeführt. Der makroskopische Befund zeigte eine muzinöse Konsistenz des Gewebes, weshalb zusätzlich auch ein Neurofibrom oder ein myxomatöser Tumor in Betracht gezogen wurde. Die erste histologische Untersuchung ergab zunächst ein kapilläres Hämangiom, beispielsweise vereinbar mit einem erworbenen, elastotischen Hämangiom. Differenzialdiagnostisch wurde auch ein Angiomyxom in Erwägung gezogen. Zur konsiliarischen Stellungnahme wurde das Präparat in die Referenzpathologie Friedrichshafen (Prof. Mentzel) eingesandt.

## Histologie

**Abb. 2:**
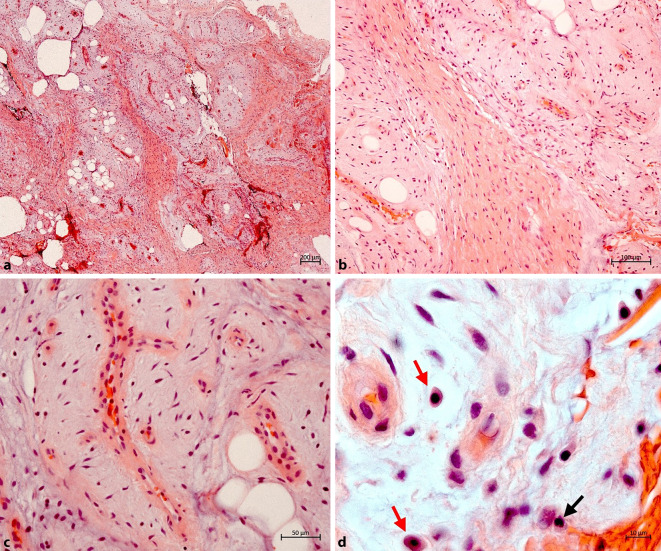
**a** Multilobulärer, dermosubkutan gelegener Tumor mit zahlreichen Gefäßen und myxoidem Stroma (Hämatoxylin-Eosin, Originalvergrößerung 2,5:1). **b** Spindelzellen und Gefäße in einem myxoiden Stroma (Hämatoxylin-Eosin, Originalvergrößerung 10:1). **c** Höhere Vergrößerung der teils plexiformen Gefäße und Spindelzellen (Hämatoxylin-Eosin, Originalvergrößerung 20:1). **d** Eingestreut fanden sich einige Mastzellen (*rote Pfeile*) und ganz vereinzelte neutrophile Granulozyten (*schwarzer Pfeil*) (Ölimmersion, Hämatoxylin-Eosin, Originalvergrößerung 63:1)

Die Histopathologie zeigte akrales Gewebe mit unauffälliger Epidermis und Nachweis einer randbildenden, multilobulär gegliederten Läsion in der darunter liegenden Dermis und Subkutis. Die Lobuli bestanden aus spindeligen, teils auch sternförmigen Zellen, die in einem myxoiden Stroma gelagert und durch fibröse Septen getrennt waren (Abb. 2) Das Stroma enthielt zahlreiche, teils plexiforme Gefäße und einige Mastzellen sowie ganz vereinzelte neutrophile Granulozyten (Abb. 2 und 3). Die immunhistochemischen Färbungen ergaben eine Positivität der spindeligen Zellen für CD34 (Abb. 4), während sich mit Alpha-Glattmuskelaktin-Antikörpern lediglich die Myoperizyten der vermehrten Gefäße markierten. Eine Expression von S100-Protein lag nicht vor.

**Abb. 3:**
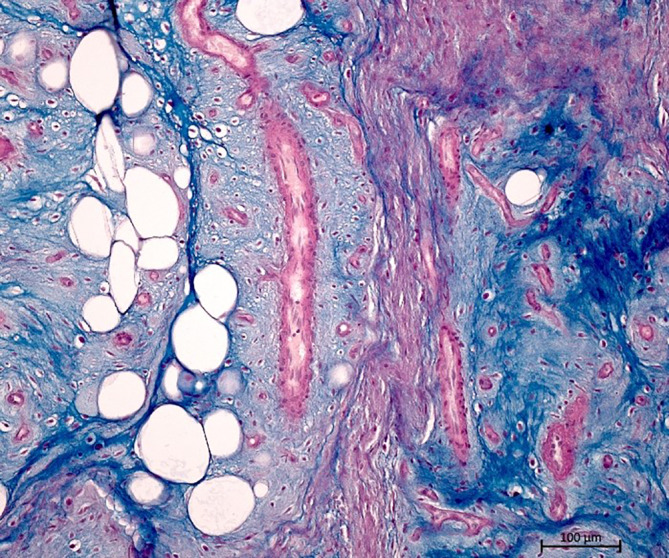
Dermal gelegener Gefäßtumor mit myxoidem Stroma (Alcian-PAS[„periodic acid-Schiff“]-Färbung, Originalvergrößerung 10:1)

**Abb. 4:**
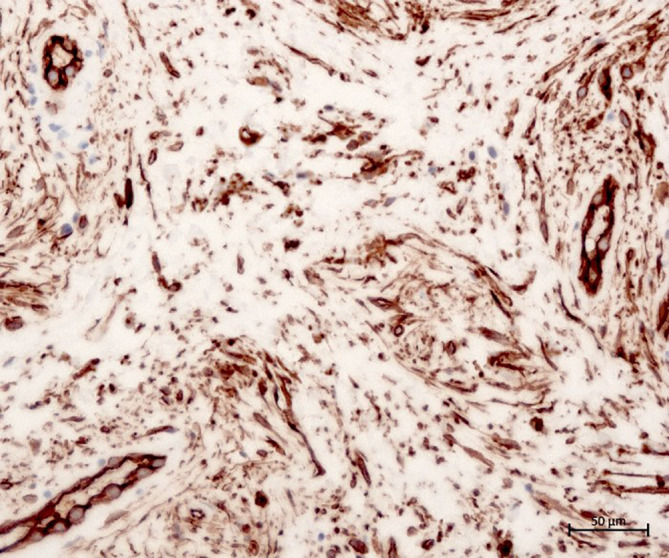
Expression von CD34 in den Spindelzellen und Gefäßendothelien (Originalvergrößerung 20:1)

## Diagnose

Superfizielles Angiomyxom

## Therapie und Verlauf

Aufgrund des Rezidivrisikos wurde eine vollständige Exzision des Tumors empfohlen, die aber vonseiten des Patienten nicht erwünscht war. Durch permanente Druckbelastung an exponierter Stelle entsteht oft eine Atrophie der unterliegenden Gewebsstrukturen, sodass weitere konservative Maßnahmen wie die Anfertigung von angepassten Schuheinlagen sinnvoll sein können.

## Diskussion

Das superfizielle Angiomyxom (SA) ist ein seltene, benigne Neoplasie aus der Familie der unbekapselten Tumoren und wurde erstmals 1985 als Teil des Carney-Syndroms beschrieben [1]. Als eigenständige Entität wurde die Neoplasie dann 1988 von Allen et al. beschrieben und später von Calonje et al. histopathologisch charakterisiert [2, 3].

Das SA tritt vorwiegend zwischen dem 30. und 50. Lebensjahr ohne Geschlechtspräferenz auf und manifestiert sich klinisch mit meist solitären, langsam wachsenden, knotigen und polypoiden Läsionen, die meist kleiner als 5 cm im Durchmesser sind.

Die häufigsten Lokalisationen sind der Rumpf, der Kopf- und Halsbereich sowie die unteren Extremitäten; beobachtet werden jedoch auch Läsionen an den oberen Extremitäten und im Genitalbereich; Letztere sind auch im Kindesalter beschrieben [2]. Das SA ist meist asymptomatisch, je nach Lokalisation kann aber auch eine funktionelle oder ästhetische Beeinträchtigung entstehen. Bei akralen SAs führt die repetitive mechanische Belastung oft zu einer Druckschmerzhaftigkeit. Wenngleich diese Neoplasien meist erworben sind, berichten manche Autoren auch über angeborene solitäre SAs am Capillitium oder in der Glandula parotis [4, 5].

Kutane Myxome können Teilsymptome syndromaler Entitäten sein, z. B. des NAME-Syndroms (Akronym für Nävus, atriales Myxom, myxoides Neurofibrom und Epheliden) bzw. des LAMB- (Akronym für Lentigines, atriales Myxom und blaue Nävi) oder des Carney-Syndroms. Als multipel auftretende Läsionen an Ohr, Augenlid oder Mamille repräsentieren SAs einen häufigen Bestandteil des Carney-Komplexes. Der Carney-Komplex ist eine seltene autosomal-dominante Erkrankung mit Auswirkungen auf die regulatorische Aktivität der Typ-Iα-Proteinkinase A [6]. Klinisch ist das Syndrom durch kutane und kardiale Myxome, Pigmentstörungen (z. B. Lentigines, blaue Nävi), eine endokrine Hyperaktivität (z. B. Cushing-Symptomatik) und neuroendokrine Tumoren gekennzeichnet. Bei unserem Patienten fand sich kein Anhalt für ein syndromales kutanes Angiomyxom.

Histologisch sind superfizielle Angiomyxome hauptsächlich dermale Läsionen von lobulärer Architektur, häufiger findet sich auch eine Ausbreitung in die Subkutis. Epitheliale Komponenten, wie z. B. epidermale Zysten oder Pilomatrixome, können auch vorhanden sein, hier wird in der Literatur über invaginierte Strukturen bzw. Kollisionstumoren diskutiert oder aber auch über eine echte Komponente des Angiomyxoms [2, 7, 8]. Die unbekapselten Neoplasien bestehen aus spindeligen bis sternförmigen, mäßig kern- und zellpolymorphen, CD34-positiven Fibroblasten in einem myxoiden Stroma, durchmischt mit Mastzellen bei ausgeprägter Vaskularisation. Stellenweise finden sich auch gruppierte mehrkernige Riesenzellen [3, 9].

Immunhistochemisch sind die plump-spindeligen Zellen meist positiv für CD34, das myxoide Stroma hingegen für Vimentin und Alpha-Glattmuskelaktin. Sehr selten wird Desmin exprimiert, hingegen sind die SAs negativ für S100-Protein, CD68, EMA (Epitheliales Membranantigen), MUC4 (Muzin 4) und AE1/3 (Zytokeratin Antikörper 1/3) [9]. Die Dermatoskopie von oberflächlichen Angiomyxomen stellt eine diagnostische Herausforderung dar. Während Green et al. das sog. „red planet sign“ als charakteristisches Merkmal beschreiben, hervorgerufen durch rote exophytische Kügelchen, beschreibt die Gruppe um Argenziano ein unspezifisches Muster der SAs: nicht pigmentierte Läsionen mit polymorphem Gefäßmuster auf einem rot-rosafarbenen Hintergrund [10, 11]. Die Ätiopathogenese der Erkrankung ist bislang nicht ausreichend geklärt. Differenzialdiagnostisch unterscheidet man sowohl benigne als auch maligne Entitäten, die meist erst histologisch und immunhistochemisch von SAs differenziert werden können (Tab. 1).

**Tab. 1:** Histologische Differenzialdiagnosen des superfiziellen Angiomyxoms

*Benigne Neoplasien*
Mukoide Pseudozyste
Oberflächliches akrales Fibromyxom
Atypischer Naevus lipomatosus
Myxoides Spindelzelllipom
Myxoides Neurofibrom
Myxoides Neurothekeom
Dermale Nervenscheidenmyxome
*Niedrigmaligne Neoplasien*
Ossifizierender fibromyxoider Tumor
Nicht ossifizierender fibromyxoider Tumor
Niedrigmalignes fibromyxoides Sarkom
Niedriggradiges Myxofibrosarkom
*Aggressive Neoplasien*
Myxoides Dermatofibrosarcoma protuberans
Aggressives (tiefes) Angiomyxom

Das SA weist einen benignen Verlauf auf, eine Metastasierung tritt nicht auf. Nicht in toto entfernte Läsionen rezidivieren jedoch in 30–40 % der Fälle, weshalb eine Gesamtexzision empfehlenswert ist [3, 12]. In kosmetisch oder funktionell anspruchsvollen anatomischen Regionen stellt die lückenlos histologisch schnittrandkontrollierte Exzision eine gewebeschonende therapeutische Option dar [13].

Zusammenfassend präsentiert die dargestellte Kasuistik eine gutartige, langsam wachsende Neoplasie. Die diagnostische Abklärung basiert insbesondere auf der charakteristischen Histopathologie und der Immunhistochemie, um das SA von anderen, teils malignen myxoiden Tumoren abzugrenzen. Obwohl es oft als eine eigenständige Entität auftritt, ist es ratsam, Syndrome wie den Carney-Komplex auszuschließen.

## Fazit für die Praxis

- Das superfizielle Angiomyxom (SA) ist ein seltener benigner Tumor und manifestiert sich meist mit solitären Knoten. Prädilektionsstellen sind der Rumpf, die Kopf-Hals-Region und die Akren, Läsionen können aber auch im Genitalbereich auftreten.
- Histopathologisch unterscheidet sich das SA von anderen myxoiden Tumoren durch die oberflächliche Lage, das Fehlen zellulärer Atypien, das Vorhandensein von stromalen entzündlichen Infiltraten und eine häufige Assoziation mit einer eingeschlossenen epithelialen Komponente.
- Therapeutisch ist eine Gesamtexzision angesichts der hohen Rezidivraten empfehlenswert. Zudem ist es ratsam, einen Zusammenhang mit dem Carney-Komplex auszuschließen.
